# Barriers in the prevention and early detection of human papillomavirus in Latin America

**DOI:** 10.1002/ijgo.70177

**Published:** 2025-04-22

**Authors:** Leopoldo Santiago Sanabria, Viviana Angélica Laverde Cardona, Yotin Ramón Pérez D, Fabrizio Abreu, María Luisina Cicerchia, Camila Cajelli, Martín de Jesús Inurreta Díaz, Mirtha Jimena Vásquez Medina, Karina Auxiliadora Amador, Gery Rosmary Ruiz Carillo

**Affiliations:** ^1^ Departamento de Ginecología Oncológica Unidad Médica de Alta Especialidad Hospital de Gineco‐Obstetricia 4 Luis Castelazo Ayala Ciudad de México México; ^2^ Departamento Ginecología y Obstetricia Hospital Universitario Clínica San Rafael Bogotá Colombia; ^3^ Departamento de Ginecología y Obstetricia Hospital Universitario Docente Central de las Fuerzas Armadas Santo Domingo República Dominicana; ^4^ Departamento de Ginecología y Obstetricia Centro Hospitalario Pereira Rossell Montevideo Uruguay; ^5^ Departamento de Ginecología y Obstetricia Hospital Dr. Cosme Argerich Buenos Aires Argentina; ^6^ Departamento de Ginecología y Obstetricia Hospital General “Dr. Manuel Gea González” Ciudad de México México; ^7^ Departamento de Ginecología y Obstetricia Hospital Nacional Arzobispo Loayza Lima Perú; ^8^ Departamento de Ginecología y Obstetricia Hospital Bautista Managua Nicaragua; ^9^ Departamento de Ginecología y Obstetricia Instituto Autónomo Hospital Universitario de Los Andes IAHULA Mérida Venezuela

## Abstract

**Objective:**

To evaluate the individual, social, cultural, health system, and structural barriers related to the human papillomavirus (HPV) in women from eight Latin American countries.

**Methods:**

A prospective, relational, and analytical study was conducted from July to August 2024 in women from eight Latin American countries: Argentina, Colombia, Mexico, Nicaragua, Peru, Dominican Republic, Uruguay, and Venezuela. Spanish‐speaking adult women who had a history of having undergone cervicovaginal cytology were included. An online survey of 34 items was designed that evaluated the different barriers to HPV medical care.

**Results:**

Overall, 1930 women were surveyed. There is a lack of education on issues related to sexual and reproductive health, as 57.3% of the participants considered HPV infection to be a sexually transmitted disease. A correlation was observed between a higher level of education and the acceptability of vaccination, as well as the taking of screening tests. It was found that 39.1% were vaccinated, which reflects the lack of resources in hospitals, where the vaccine is not available to all women, a similar situation to the screening test.

**Conclusion:**

There are still many barriers to HPV medical care due to the cultural roots that exist in the region. It is important to identify them and create strategies that allow equality and accessible medical care for all women, in order to eradicate cervical cancer, which is one of the great pending issues on the gynecologist's agenda.

## 1 INTRODUCTION

Cervical cancer is one of the main public health problems faced in Latin America which is affected by different sectors such as education, health, and public policies. In Latin America, the prevalence is estimated to be around 32%.^1^

The Global Cancer Observatory (GLOBOCAN) reported that in 2024, cervical cancer ranks eighth in women's cancer in terms of incidence and ninth in terms of mortality. Latin America ranks third in the world with regard to incidence and fourth with regard to both mortality and prevalence. In the last year, this region registered the following percentages worldwide: 9.5% of new cases, 9.6% in mortality, and 10.2% in prevalence.^2^

The WHO and the International Federation of Gynecology and Obstetrics (FIGO) are committed to eradicating cervical cancer. Their strategy is founded on three pillars: 90% vaccination—in girls under 15 years of age; screening—in 70% of women at ages 35 and 45 years; and treatment—of 90% of women with cervical cancer precursor lesions and 90% of women with invasive cancers.^3^, ^4^

Despite the great efforts made in terms of public health policies, low‐ and middle‐income countries (LMICS) face multiple barriers, which include lack of resources, costs, lack of knowledge about human papillomavirus (HPV) infection, acceptability of vaccination, cultural stigmas, and accessibility and affordability in screening studies.^5^

The lack of education and awareness, as well as individual and collective beliefs generate a negative impact on the prevention initiative. Cultural roots tend to be strong in some Latin American societies, especially in sexual and reproductive health, which are still considered taboo topics.^6^, ^7^ Nonetheless, recently education through schools has generated greater acceptability in vaccination and screening studies.^8^, ^9^

In the last decade, the region made great progress, such as the introduction of vaccination in 44 national vaccination programs, but following the COVID‐19 pandemic, there has been a decline in vaccination. Currently, the strategies focus mainly on catch‐up vaccination campaigns, in order to retain what has been achieved.^10^

It is estimated that 90% of cervical cancers around the world occur in LMICs.^11^ The situation seems unlikely to change in the future, which is why there is a need to establish solid strategies that reinforce preventive attitudes, and implement promising advances, such as therapeutic vaccination.^12^

The objective of the present study was to evaluate the individual, social, cultural, health system, and structural barriers related to HPV in women from eight Latin American countries.

## 2 MATERIALS AND METHODS

A prospective, observational, relational, and analytical study was carried out in women from eight Latin American countries: Argentina, Colombia, México, Nicaragua, Peru, Dominican Republic, Uruguay, and Venezuela. A survey was conducted over a period of 2 months from July 1 to August 31, 2024. Participant consent was obtained before answering the survey, which included a message containing the objectives of the study, data protection, and confidentiality. The research was conducted in accordance with the Declaration of Helsinki. Approval was obtained from the Ethics and Research Committee of the Unidad Medica de Alta Especialidad 4 (ID I24‐0051).

An online survey was applied to adult women from the participating countries. Women who did not speak Spanish, who had never attended a cervicovaginal cytology test, or who did not have the technology to access the online survey were excluded. Only one response per participant was allowed and duplicate questionnaires were removed.

An instrument was designed after an extensive review of the literature and other questionnaires about the barriers to, knowledge of, and acceptance of HPV prevention and detection in women.^13^, ^14^ Questions were adapted to the Latin American women, and the survey was conducted in Spanish.

The final survey included 34 questions, which inquired about sociodemographic data and barriers to medical care for HPV infection. All questions included were designed with a mandatory response, closed, and in multiple‐choice format. The Google Forms program (Google LLC, Mountain View, CA, USA) was used to create the instrument.

Statistical analysis was performed with the SPSS statistical program version 29.0.2.0 (IBM, Armock, NY, USA). Descriptive statistics were used. The chi‐squared test and Fisher test were used for the analysis. A *P*‐value of less than 0.05 was considered to reflect statistically significance. Each survey question was analyzed individually.

## 3 RESULTS

**FIGURE 1:**
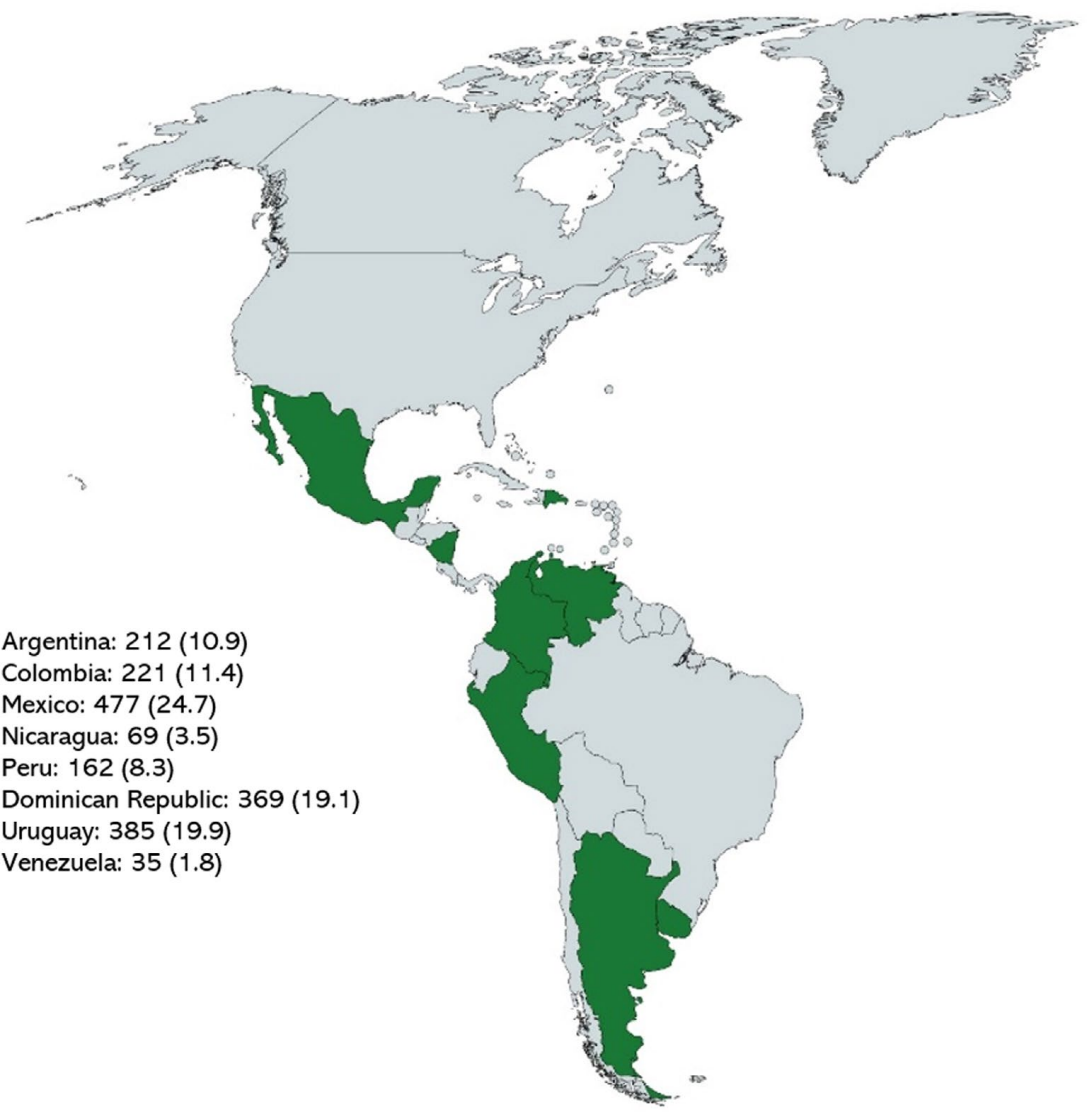
Participating women by country (*n* [%]).

In all, 1930 number women completed the survey, in eight Latin American countries. The distribution by country was as follows (Figure 1): Argentina (10.9%), Colombia (11.4%), Mexico (24.7%), Nicaragua (3.5%), Peru (8.3%), Dominican Republic (19.1%), Uruguay (19.9%), and Venezuela (1.8%).

**FIGURE 2:**
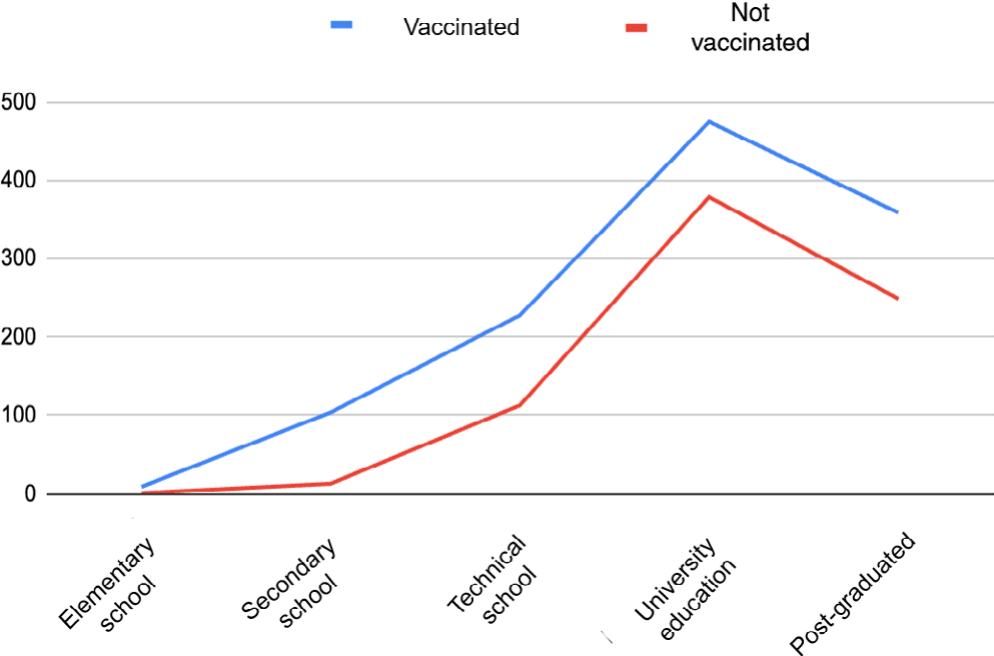
Education level and acceptance of human papillomavirus (HPV) vaccination.

The sociodemographic characteristics of the participants are outlined in Table 1. The average age was 37.04 years. The majority (854 [44.2%]) had a college degree, and 1738 (90.1%) of the women surveyed lived in cities considered as urban areas. It was reported that 1051 (54.4%) women had private health insurance, 457 (23.6%) had public insurance, 355 (18.3%) had both types of social security, and 67 (3.4%) did not have health insurance. Figure 2 shows the correlation between the level of education and the acceptance of HPV vaccination, which was higher in women with university education.

**TABLE 1:** Sociodemographic characteristics.

	Total (*n* = 1930) (%)
Age	
Average age (y)	37.04^a^
18–26	243 (12.6)
27–35	844 (43.7)
36–50	545 (28.2)
51–60	181 (9.4)
>61	117 (6.1)
Country	
Argentina	212 (10.9)
Colombia	221 (11.4)
Mexico	477 (24.7)
Nicaragua	69 (3.5)
Peru	162 (8.3)
Dominican Republic	369 (19.1)
Uruguay	385 (19.9)
Venezuela	35 (1.8)
Education level	
Elementary School	10 (0.5)
Secondary School	117 (6.06)
Technical School	341 (17.6)
University education	854 (44.2)
Post‐graduated	608 (31.5)
Living area	
Rural	192 (9.9)
Urban	1738 (90.1)
Health insurance	
Private	1051 (54.4)
Public	457 (23.6)
Both	355 (18.3)
No health insurance	67 (3.4)

*Note*: Data are presented as *n* (%) unless noted otherwise.

^a^ Value given as average.

The data regarding knowledge about HPV screening and vaccination are given in Table 2. A total of 1106 (57.3%) women reported that HPV infection is a sexually transmitted disease, and 1765 (91.4%) were aware that HPV infection could cause some type of cancer. Similarly, 1664 (86.2%) thought that any woman can be exposed to this virus during adult life. Regarding vaccination, 1349 (69.8%) women reported that even infected people can receive the vaccine, and 1665 (86.2%) said that it can prevent a certain type of cancer. A total of 1891 (97.9%) considered that screening tests are important for the detection of HPV, but about half of them (864 [44.7%]) did not know or had not heard about molecular tests for the detection of this virus.

**TABLE 2:** Individual barriers: Knowledge about HPV screening tests and vaccination.

Question	Total (*n* = 1930)	*P*‐value	95% CI
Is HPV infection transmitted sexually?			
Yes	1106 (57.3)	0.010	1.60–8.20
No	655 (33.9)		
No sure	169 (8.7)		
Can HPV infection cause any type of cancer?			
Yes	1765 (91.4)	0.150	−1.30–7.30
No	27 (1.3)		
No sure	138 (7.1)		
Do you think that any adult woman can be exposed to HPV?			
Yes	1664 (86.2)	0.040	2.50–11.10
No	156 (8.08)		
No sure	110 (5.6)		
Should people infected with HPV be vaccinated?			
Yes	1349 (69.8)	0.130	−1.30–3.00
No	216 (11.1)		
No sure	365 (18.9)		
Does the HPV vaccine help prevent any type of cancer?			
Yes	1665 (86.2)	0.010	1.10–8.30
No	58 (3.0)		
No sure	207 (10.7)		
Are screening studies important for HPV detection?			
Yes	1891 (97.9)	<0.001	3.10–14.50
No	39 (2.09)		
Do you know or have you heard about the HPV PCR for HPV test?			
Yes	1066 (55.2)	0.020	2.10–12.00
No	864 (44.7)		

*Note*: Data are presented as *n* (%).

Abbreviations: CI, confidence interval; HPV, human papillomavirus; PCR, polymerase chain reaction.

**FIGURE 3:**
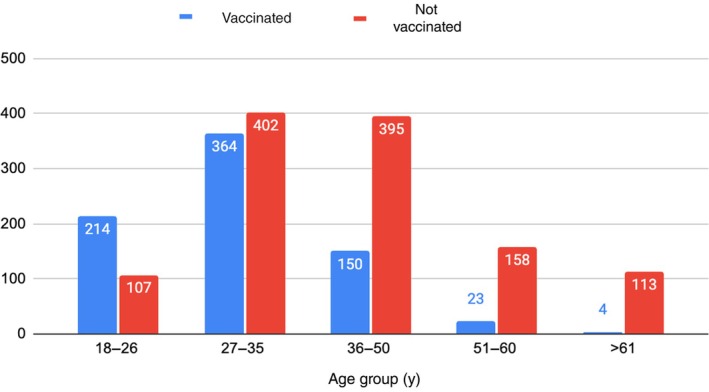
Age groups and vaccination rate.

The evaluation of acceptability regarding vaccination is shown in Table 3. A total of 755 (39.1%) women reported being vaccinated, and most vaccinated women (288 [14.9%]) reported having received two doses. The most frequently administered vaccine was the quadrivalent vaccine, in 254 (13.2%) women, although 368 (19.1%) of the vaccinated women did not remember which vaccine they had received. Figure 3 shows that the age group with the highest vaccination rate was 27–35 years; however, a trend of lower vaccination at older ages is observed. It was documented that 1141 (59.1%) women considered that there are social, cultural, or religious barriers that do not allow HPV vaccination. Among the participants, 1508 (78.1%) considered that it is equally important for boys and men to receive the vaccine. When asked about the importance of promoting vaccination campaigns for boys and girls between 9 and 15 years of age, 1659 (86%) women considered it important. Most of the participants (1833 [95%]) would recommend vaccination among their family and friends.

**TABLE 3:** Individual, social, and cultural barriers towards the acceptance of human papillomavirus (HPV) vaccination.

Question	Total (*n* = 1930)	*P‐*value	95% CI
Are you vaccinated against HPV?			
Yes	755 (39.1)	0.030	1.10.‐3.50
No	1175 (60.9)		
How many doses did you receive?			
1	102 (5.3)	0.001	2.30–5.10
2	288 (14.9)		
3	268 (13.9)		
No sure	97 (5.0)		
Does not apply	1175 (60.9)		
What vaccine did you receive?			
Bivalent (VPH16‐18)	56 (2.9)	0.030	3.20–10.10
Quadrivalent (VPH6,11,16,18)	254 (13.2)		
Nonavalent (VPH6,11,16,18, 31,33,45,52,58)	77 (4.0)		
No sure	368 (19.1)		
None	1175 (60.9)		
Do you think there are social, cultural, or religious factors that do not allow HPV vaccination?			
Yes	1141 (59.1)	0.070	−1.30–3.20
No	789 (40.9)		
Is it important for boys and men to receive the HPV vaccine?			
Yes	1508 (78.1)	0.010	2.10–4.50
No	169 (8.8)		
No sure	253 (13.1)		
Is it important to promote vaccination campaigns in boys and girls between 9 and 15 years old?			
Yes	1659 (86.0)	0.003	3.20–7.10
No	77 (4.0)		
No sure	194 (10.1)		
Would you recommend HPV vaccination to your family and friends?			
Yes	1833 (95.0)	0.020	1.70–11.30
No	97 (5.0)		

*Note*: Data are presented as *n* (%).

Abbreviation: CI, confidence interval.

The evaluation of the structural, cultural, and health system barriers is shown in Table 4. Regarding the structural barriers, the participants reported an average travel time of 33.22 minutes to their local hospitals. A significant number of women (1460 [75.6%]) reported that some healthcare personnel had provided them with information about HPV. Less than half (798 [41.3%]) reported that vaccines are available in their local hospitals.

**TABLE 4:** Cultural, health system, and structural barriers regarding the prevention, diagnosis, and timely treatment of human papillomavirus (HPV).

Question	Total (*n* = 1930)^a^	*P*‐value	95% CI
Has healthcare staff told you about HPV infection?			
Yes	1460 (75.6)	0.001	3.40–7.60
No	265 (13.7)		
No sure	205 (10.6)		
Are there campaigns that carry out cervicovaginal cytology in your area?			
Yes	1127 (58.4)	0.049	2.10–5.20
No	410 (21.2)		
Have never heard of it	393 (20.4)		
Are there HPV vaccination campaigns in your area?			
Yes	686 (35.5)	0.006	3.40–15.30
No	803 (41.6)		
Have never heard of it	441(22.8)		
Are HPV vaccines available in public institutions in your country?			
Yes	798 (41.3)	0.070	−1.20–3.40
No	436 (22.6)		
No sure	696 (36.1)		
Have you received your cervicovaginal cytology results?			
Yes, I have always received them	1458 (75.5)	<0.001	2.50–5.60
No, I have only received them on some occasions	259 (13.4)		
No, I have never received them	59 (3.05)		
Does not apply	154 (7.9)		
Average time to receive results from cervicovaginal cytology (d)	23.6		
Do you think the cervicovaginal cytology test is affordable?			
Yes	1575 (81.6)	<0.001	2.5–12.3
No	188 (9.7)		
Don't know the price	167 (8.7)		
Have you obtained an altered result in your cervicovaginal cytology?			
Yes	615 (31.9)	0.020	2.10–3.40
No	1252 (64.9)		
No sure	63 (3.3)		
What was the diagnosis of your altered cervicovaginal cytology?			
Atypical squamous cells of undetermined significance	82 (4.2)	<0.010	1.20–4.20
Atypical glandular cells of undetermined significance	46 (2.4)		
Low‐grade intraepithelial lesion	285 (14.8)		
High‐grade intraepithelial lesion	67 (3.5)		
Carcinoma in situ	25 (1.3)		
No sure	110 (5.7)		
Does not apply	1315 (68.1)		
If yes, were you referred to a tertiary center due to the altered result?			
Yes	201 (10.4)	0.020	2.40–5.30
No	529 (27.4)		
Does not apply	1200 (62.2)		
Have you ever had a PCR test for HPV?			
Yes	541 (28)	0.003	2.60–7.30
No	1009 (52.3)		
No sure	380 (19.7)		
If you have never done it, what was the reason?			
Have never heard of it	569 (29.4)	0.015	3.30–7.80
Health insurance does not include it	303 (15.6)		
Not interested in doing it	112 (5.8)		
Due to age, doctor did not prescribe it	25 (1.2)		
Does not apply	921 (47.7)		
Do you think there is discrimination against people with HPV infection by healthcare personnel?			
Yes	396 (20.5)	0.020	2.20–6.40
No	1534 (79.4)		
Do you think there is discrimination by family and friends of people infected with HPV?			
Yes	910 (47.2)	0.010	3.10–6.30
No	1020 (52.8)		
Average transfer time to the nearest hospital (min)	23.6		

Abbreviations: CI, confidence interval; PCR, polymerase chain reaction.

^a^ Data are presented as *n* (%) unless noted otherwise.

On the other hand, regarding cultural barriers, 396 (20.5%) participants reported that there is discrimination in medical care by health personnel against women who have been diagnosed with HPV, and the frequency of discrimination by family members and friends was higher, as reported by 910 (47.2%) women.

Regarding the barriers in the health system, they were questioned about the existence of campaigns that promote cervicovaginal cytology, to which 1127 (58.4%) women reported that they exist in their localities, but the frequency was lower regarding vaccination campaigns, where only 686 (35.5%) women said that these exist in their communities. A total of 615 (31.9%) women had received an inaccurate result in their screening test, and the most frequently reported of these was low‐grade squamous intraepithelial lesion, in 285 (14.8%) women. Only 201 (10.4%) were referred to a tertiary center. About half (1009 [52.3%]) of the women said that they had undergone a polymerase chain reaction (PCR) test, and the main reason for not having this test, according to 541 (28%) women, was ignorance about its existence.

It is important to note that there were 58 women who had never been screened for cervical cancer. There were not included in the analysis as not all barriers could be assessed, surveys were not completed, and they tended to be different, such as language barriers, accessibility to hospitals, and religious beliefs. The average age of this group was 57 years, older than any group that had ever been screened, their education level was lower, with almost all attending only primary school, and they lived in rural areas with strong cultural beliefs, belonged to indigenous groups, and had public or no health insurance. None of them were vaccinated and they had never heard about HPV infection.

## 4 DISCUSSION

The present study documented that there are different areas of opportunity in awareness, prevention, and medical care regarding HPV infection in Latin America. It was shown that the barriers begin with individual factors such as lack of knowledge, and are combined with existing gaps in health policies, which are meant to guarantee optimal primary and secondary prevention. These barriers are grouped into five categories: individual, cultural, social, those related to the health system, and structural.^15^, ^16^

The main individual barrier is the lack of knowledge and information about prevention and screening, the value of which is usually underrated. Sociodemographic factors showed that women with more education are more likely to attend screening tests more frequently and have greater acceptability of vaccination. As shown in the present study, women with university education had greater acceptability of the vaccine.^17^ A general lack of knowledge regarding HPV infection was also documented in this study. Many women are not aware that HPV is a sexually transmitted infection and have not heard about effective screening tests, such as molecular testing. Most of participants were aware that every adult woman is at risk of becoming infected during her life, which is why vaccines are a preventive action. However, there are still barriers to vaccination.

Other authors have noted that the lack of knowledge and misinformation among adolescent women is an important barrier to vaccination. However, through educational interventions carried out in secondary schools, with both students and parents in other LMICs, it has been possible to reinforce awareness of cervical cancer prevention and increase vaccination rates.^18^ The present study showed that the only age group with a higher rate of vaccinated than unvaccinated women is 18–26 years old, given that the initiatives in these countries are recent.

On the other hand, social barriers tend to include disapproval of screening tests as HPV is believed to be related to infidelity and promiscuity, and this is reflected in a lack of support from the partner and family. Community education is essential to break down stigmas and increase awareness around this sexually transmitted infection, in both genders.^19^ More than half of the participants thought that there is a lack of social support that does not allow them to seek medical attention.

Therefore, strategies that empower women and make them feel involved in the prevention process must be reinforced. A viable option has been the implementation of self‐testing for HPV, which has been satisfactory in increasing screening in settings where there is gender inequality, especially in communities with traditional values.^20^

Cultural barriers are the most difficult to modify. Historically, sexual and reproductive health has been stigmatized, which prohibits practicing a culture of prevention as these topics are still taboo in some societies. Some of the most reported barriers are the fear of being rejected by the community and the shame caused by having to undress in front of healthcare personnel.^21^ Nearly half of the participating women reported that there is still discrimination by family members and friends against women who have been diagnosed with HPV. Many prefer not to seek medical attention due to fear of rejection. This problem has even been reported by healthcare personnel, which makes it even more difficult.

It is the responsibility of healthcare providers to change perceptions around cervical cancer prevention. The benefits they entail should be emphasized and it should be noted that it is a pathology that can be treated in its pre‐invasive stage, before it manifests as cancer. Religious leaders should be involved in encouraging women to become aware of their health and to seek preventive services.

Regarding the health system, there are gaps in public policies that guarantee sufficient resources to carry out effective prevention, which includes trained healthcare personnel. A public health strategy that has been successful in increasing vaccination coverage in Latin America is the one proposed in the ESCUDDO trial carried out in Costa Rica. This research documented that the administration of a single dose for HPV seems to be non‐inferior to traditional schemes, showing acceptable antibody titers, especially for HPV16 and HPV18.^22^ Effective public policies that promote universal vaccination should be supported. In an ideal world, vaccination should be free and without gender distinction, to obtain greater coverage.^23^ A significant percentage of the participants continue to believe that children and men should not be vaccinated, given that the campaigns have only tended to focus on women. There is a great challenge in increasing vaccination coverage, given that in the present study it was reported that 60.9% of the participants were not vaccinated, which correlates with the vaccination rates published in Latin America.

The structural barriers consist of the accessibility and affordability of screening tests and vaccination, as well as travel times to hospitals that provide quality care, which includes education to patients.^24^ This study reflected that transfer times continue to be long and hospitals are not adequately equipped, as there is a shortage of vaccines and resources for adequate screening in public institutions. There are also no organized campaigns, for either vaccination or screening, to cover these gaps in public hospitals. In addition, delivery times for cervicovaginal cytology results are long, on average 23.60 days, which is an indicator that can be improved.

The present study has some limitations. Although the sample size was adequate, in some countries there were few responses, which may be attributable to social causes and poor access to online resources. However, one of the main strengths was the evaluation of different indicators in Latin American groups that had not previously been studied. Regarding the group that had never been screened for cervical cancer, it would be interesting to evaluate them in another study, as they face other types of barriers, including language, because in Latin America there are many indigenous groups that speak languages other than Spanish and almost all of them have never heard about cervical cancer screening or even HPV infection.

In conclusion, there are different barriers in medical care related to HPV; some can be difficult to modify, such as cultural or religious values. Health personnel must focus on those that can be modified, such as the promotion of screening tests and vaccination. The decision to behave in a certain way, such as participation in HPV prevention, is not unidirectional, but depends on several factors such as obtaining objective information, which will be influenced by behavioral beliefs dictated by society, making the acceptance of both screening studies and vaccination complex.

Therefore, gynecologists must act as allies in providing true information about sexually transmitted infections. This will likely motivate many women to make important decisions regarding the prevention of pathologies associated with HPV. No effort is useless, if we keep in mind our great commitment, which is the elimination of cervical cancer, a common preventable disease.

## AUTHOR CONTRIBUTIONS

LSS, VALC, YRPD, and MJID contributed to the conception and design of the protocol. LSS, VALC, FA, CC, MLC, KAA, and GRRC designed the survey after reviewing the literature. LSS, VALC, YRP, and MJVM contributed to the acquisition of the data. VALC and MJVM performed the statistical analysis. All authors collaborated equally in the conduction of the surveys. All authors approved the final version of the manuscript for publication.

## FUNDING INFORMATION

This research received no specific grant from any funding agency in the public, commercial, or not‐for‐profit sectors.

## CONFLICT OF INTEREST STATEMENT

The authors have no conflicts of interest.

## PLAGIARISM

All authors confirm that there is no instance of plagiarism in our manuscript.

## Data Availability

Research data are not shared.
